# Ulnar Longitudinal Deficiency: A Case Report

**DOI:** 10.7759/cureus.40111

**Published:** 2023-06-08

**Authors:** Sara Velasquez Restrespo, Victor N Oboli, Dyuti Kumar, Catalina Marino-Villamizar, Shefali Khanna

**Affiliations:** 1 Pediatrics, New York City (NYC) Health + Hospitals/Lincoln, Bronx, USA; 2 Surgery, St. George's University School of Medicine, St. George's, GRD; 3 Pediatric Medicine, New York City (NYC) Health + Hospitals/Lincoln, Bronx, USA

**Keywords:** rotational osteotomies, thrombocytopenia with absent radii (tar) syndrome, ulna bone, upper extremity deformities, congenital, hemimelia, ulnar hemimelia, radial longitudinal deficiency, ulnar longitudinal deficiency

## Abstract

*Ulnar longitudinal deficiency *(ULD) is a rare skeletal condition marked by the partial or complete failure of the formation of the ulna. This rare condition is often associated with fixed flexion deformity, radial head subluxation, complex carpal, metacarpal, and digital abnormalities. Most presentations are male-preponderant and right-sided. Different classifications have described ULD. Usually, the condition is not associated with systemic findings; however, detailed physical examination and radiologic evaluations are crucial for assessing and managing affected patients. We report a rare case of ULD in an 11-month-old female infant with congenital absence of the left ulna, four digits, and a postaxial hypoplastic finger.

## Introduction

Ulnar longitudinal deficiency (ULD), also known as "congenital ulnar hemimelia," "postaxial longitudinal deficiency of the upper limb," "ulnar club hand," or "ulnar ray deficiency," is a rare sporadic skeletal condition marked by a partial or complete failure of formation of the ulna bone. ULD is a part of congenital longitudinal deficiencies, a spectrum of abnormalities that affect the upper extremities, with radial longitudinal deficiency being more common than others. Radial longitudinal deficiency affects 1:30,000 live births, while ULD affects one to two in 100,000 children [1-3]. ULD is primarily found in males rather than females, with a male-to-female ratio of 3:2. Most presentations (70%) are right-sided and unilateral, with ulnar deviation of the hand and a shortened forearm [1-4].

## Case presentation

We present an 11-month-old female infant, a recent immigrant from Colombia, delivered small for gestational age with congenital absence of the left ulna bone at 37 weeks gestational age through emergency cesarean section due to maternal preeclampsia and hemolysis, elevated liver enzyme, low platelet count (HELLP) syndrome. There was no history of teratogenic medication ingestions during the perinatal period but a previous miscarriage from an unknown etiology.

**Figure 1:**
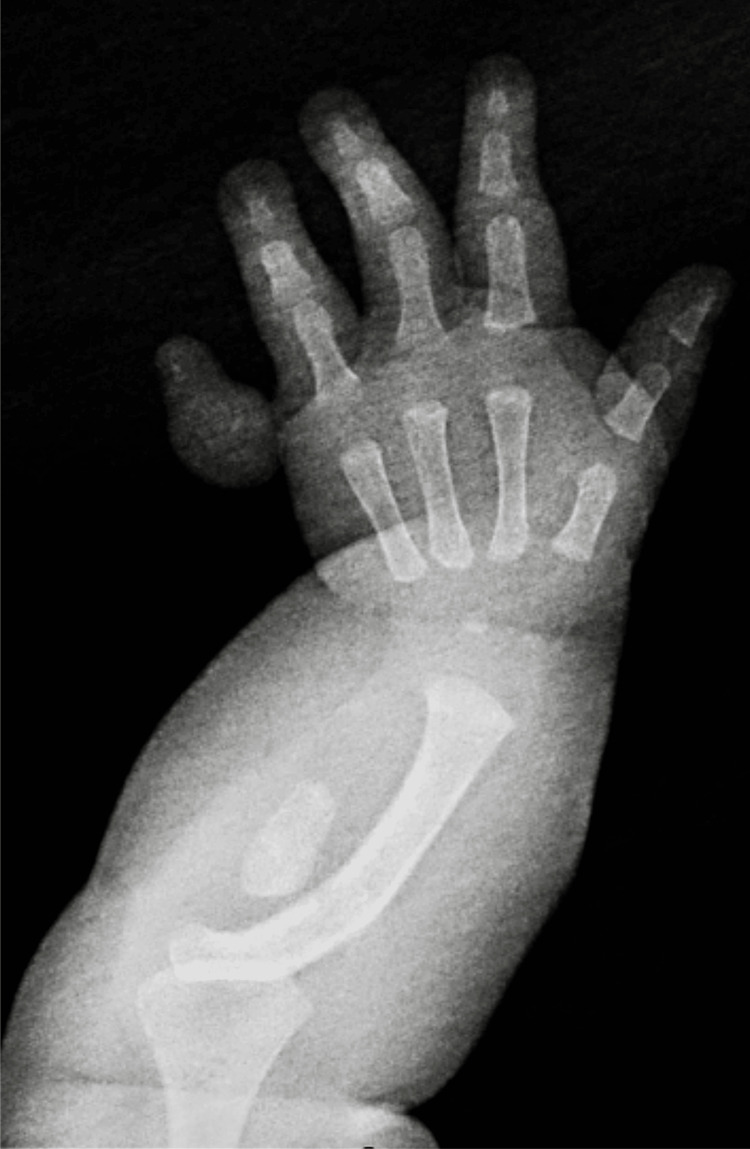
Left forearm and hand radiograph depicting a small ovoid bone in the expected location of the ulna without articulation to the humerus or wrist.

**Figure 2:**
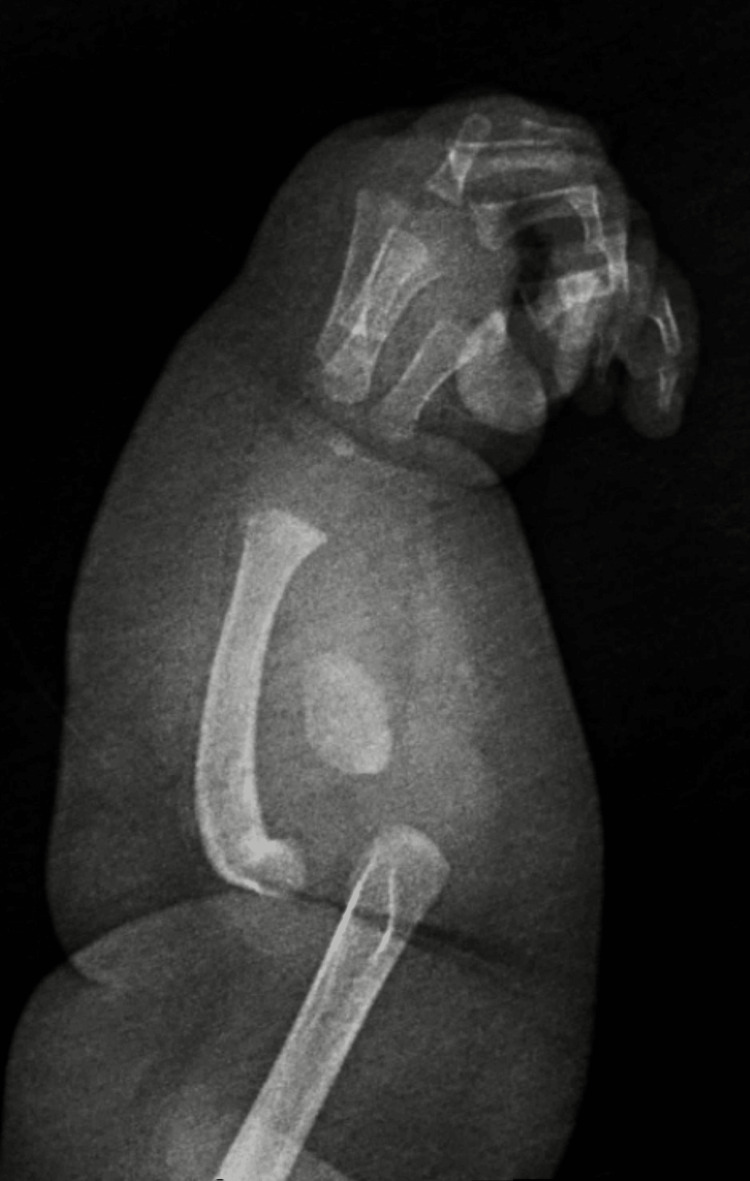
Left forearm and hand radiograph showing moderate bowing of the radius and frank dislocation with respect to the humerus.

Our patient presented to the ambulatory clinic for a health supervision visit at five months of age. Her anthropometric parameters (weight: 8.22 kg, height: 67 cm, head circumference: 42 cm) and developmental milestones were appropriate for her age, and her immunizations were up-to-date. At that visit, her physical examination was pertinent for candidal diaper rash and a deformed, hypoplastic left upper extremity (LUE) with four digits and a postaxial hypoplastic finger. Her mother noted no history of abnormal bleeding, cardiac problems, recurrent infections, or delayed wound healing since birth. There was no family history of genetic disorders, skeletal abnormalities, or physical findings of VACTERL (vertebral defects, anal atresia, cardiac defects, tracheoesophageal fistula, renal anomalies, and limb deformities) association. However, routine immigrant screening showed a positive tuberculin skin test of 20 mm (reference range: <5 mm), but she was without any signs and symptoms of active tuberculosis. There was no exposure to contacts with chronic cough or tuberculosis; there was no fever, cough, poor feeding, growth faltering, or palpable enlarged lymph node swellings. Chest, shoulder, humeral, and hip X-rays were normal. The left upper limb X-ray was significant for a small ovoid bone in the location of the ulna and fifth metatarsal with moderate bowing of the radius (Figures 1, 2). Other investigations were unremarkable, including a hip ultrasound, complete blood count, and hepatic function panel. She was admitted to begin treatment for latent tuberculosis. Subsequently, she got discharged to be followed up by the infectious disease, genetics, and orthopedic outpatient teams for further management.

## Discussion

ULD is a rare sporadic skeletal condition. It is characterized by a longitudinal deficiency at the posterior axis of the upper limb where the ulna bone is situated. Its etiology is frequently related to the deficiency of the *Sonic Hedgehog* gene pathway responsible for the development of ulnar-sided forearm structures and four ulnar-sided digits. This *Sonic Hedgehog* gene deficiency explains the thumb abnormalities that occasionally accompany ulna dysplasias [3-5].

ULD is usually isolated and not commonly associated with any systemic conditions. However, specific syndromes like limb/pelvis hypoplasia/aplasia syndrome, Weyer's oligodactyly syndrome, and ulnar-mammary syndrome have ULD as an associated feature [6]. It is frequently associated with other musculoskeletal conditions such as proximal femoral focal deficiency, radial head subluxation, syndactyly, oligodactyly, or congenital scoliosis. Approximately 90% of patients with ULD have missing digits, 70% with thumb abnormalities, and 30% present with syndactyly [2,7,8]. Another clinical condition to consider when evaluating patients with hypoplastic upper limbs is thrombocytopenia absent radius (TAR) syndrome. TAR syndrome is characterized by bilateral absent radii and transient thrombocytopenia with an associated cow's milk allergy. TAR differs from ULD by the presence of thumbs without missing digits, and 17-24% of patients with TAR have cardiac and genitourinary anomalies [9].

Different classifications have described ULD depending on several factors. Ogden et al. classified the anomalies based on the severity of the ulnar deformity. Bayne classification has been widely used and classifies ULD into four types (type 1: ulnar hypoplasia with intact proximal and distal epiphysis; type 2: partial ulnar aplasia, which is the most common type; type 3: complete ulnar absence with carpal and digital deficiencies; and type 4: radiohumeral synostosis) [10]. Our patient most likely had type 2 and 3 ULD features with an absent fifth digit. Ogino and Kato further included the deficiency of fingers on the ulnar side to contribute to the classification system [11]. Cole and Manske incorporated the thumb or first web deformity into the pre-existing ulnar ray deficiency [7]. Havenhill et al. modified Bayne classification, including ulna hand and carpal deficiencies without forearm or elbow involvement [12].

Treatment of ULD depends on the severity of the malformation and targets improvement of limb function and overall quality of life. Management involves a clinical geneticist consultation and referral to the orthopedic and plastic surgical teams for evaluation and surgery if required. Treatment would be either operative or nonoperative, depending on the elbow and forearm anomalies classification. Nonoperative treatments include early stretching and splinting at a young age. Surgical treatments are recommended after the second year of life, including tissue distractions, syndactyly releases, deepening of first web space, opponensplasty, metacarpal rotational osteotomies, and pollicization [3,7].

## Conclusions

ULD is a rare form of congenital longitudinal deficiency associated with digital abnormalities, as presented in our case report of an 11-month-old female infant with ULD type 3 according to Bayne classification with associated oligodactyly and postaxial hypoplastic finger. Therefore, prompt diagnosis by detailed history, complete physical examination, and early referral to the surgical teams are paramount when these patients are encountered to prevent complications such as contractures, improve functionality, and avoid secondary psychological disruption to the patients and their families.
